# *KIF5A* p.Pro986Leu Risk Variant and Accelerated Progression of Amyotrophic Lateral Sclerosis

**DOI:** 10.1002/acn3.70059

**Published:** 2025-04-25

**Authors:** Arianna Manini, Rosario Vasta, Alberto Brusati, Francesco Scheveger, Silvia Peverelli, Alessio Maranzano, Alberto Doretti, Francesco Gentile, Eleonora Colombo, Maura Brunetti, Cristina Moglia, Antonio Canosa, Umberto Manera, Maurizio Grassano, Davide Gentilini, Stefano Messina, Federico Verde, Claudia Morelli, John E. Landers, Bryan J. Traynor, Adriano Chiò, Vincenzo Silani, Andrea Calvo, Antonia Ratti, Nicola Ticozzi

**Affiliations:** ^1^ Department of Pathophysiology and Transplantation, “Dino Ferrari” Center Università degli Studi di Milano Milan Italy; ^2^ Rita Levi Montalcini Department of Neuroscience University of Turin Turin Italy; ^3^ Neuromuscular Diseases Research Section National Institute on Aging Bethesda Maryland USA; ^4^ Department of Brain and Behavioral Sciences Università degli Studi di Pavia Pavia Italy; ^5^ Neurology Residency Program Università degli Studi di Milano Milan Italy; ^6^ Department of Neurology‐Laboratory of Neuroscience IRCCS, Istituto Auxologico Italiano Milan Italy; ^7^ Biology of Myelin Unit, Division of Genetics and Cell Biology IRCCS San Raffaele Scientific Institute Milan Italy; ^8^ Neurology Unit 1U “City of Health and Science” University Hospital Turin Italy; ^9^ Institute of Cognitive Sciences and Technologies, National Council of Research Rome Italy; ^10^ Bioinformatics and Statistical Genomic Unit IRCCS Istituto Auxologico Italiano Milan Italy; ^11^ Department of Neurology University of Massachusetts Chan Medical School Worcester Massachusetts USA; ^12^ Neuroscience Program, Morningside Graduate School of Biomedical Sciences University of Massachusetts Chan Medical School Worcester Massachusetts USA; ^13^ Department of Neurology Johns Hopkins University Medical Center Baltimore Maryland USA; ^14^ Therapeutics Development Laboratory National Center for Advancing Translational Sciences Rockville Maryland USA; ^15^ Reta Lila Weston Institute, Institute of Neurology University College London London UK; ^16^ Department Medical Biotechnology and Translational Medicine Università degli Studi di Milano Milan Italy

**Keywords:** alleles, amyotrophic lateral sclerosis, disease progression, KIF5A, motor neurons

## Abstract

This study explored the impact of *KIF5A* rs113247976 (p.Pro986Leu), a risk allele for amyotrophic lateral sclerosis (ALS), on phenotypic variability in two Italian ALS cohorts (discovery, *n* = 865; replication, *n* = 1174). The minor allele (T) frequency was 0.015. No patients were homozygous (TT), allowing comparison between wild type and heterozygous carriers only. Heterozygous carriers showed faster disease progression (ALSFRS‐R preslope). Findings were validated across both cohorts. Multiple linear regression identified p.Leu986 and age at onset as ALSFRS‐R preslope predictors. In conclusion, heterozygous p.Leu986 in *KIF5A* is associated with faster ALS progression, supporting its consideration for genetic screening in clinical trials.

## 1 Introduction

Amyotrophic lateral sclerosis (ALS) is a neurodegenerative disorder affecting upper and lower motor neurons, resulting in progressive loss of independence due to muscular weakness and atrophy, and death often due to respiratory insufficiency. ALS is mainly sporadic, although in 5%–10% of cases a positive family history of motor neuron disease is reported, with high genetic heterogeneity [1].

In 2018, *KIF5A*, a member of the kinesin molecular motor superfamily expressed in neurons and involved in axonal transport, was identified as an ALS‐associated gene [2]. In particular, the coding rs113247976 single nucleotide polymorphism (SNP) (p.Pro986Leu) was significantly associated with ALS risk, while an excess of loss‐of‐function mutations in the C‐terminal cargo‐binding domain was observed in ALS patients compared to controls [2]. While most KIF5A is typically cytosolic, unbound from cargo, and inhibited by head‐tail association, ALS‐associated mutations create a constitutively active kinesin lacking autoinhibition, leading to increased microtubule binding, altered dynamics, and accumulation in distal neurites, and altered axonal transport [3]. Furthermore, ALS‐related *KIF5A* mutations alter KIF5A protein and RNA interactions, affecting RNA processing and cellular stress response pathways [3].

Interestingly, rare variants in other functional domains have already been associated with distinct neurodegenerative diseases (i.e., Hereditary Spastic Paraplegia type 10, Charcot–Marie‐Tooth type 2, intractable myoclonus) [4, 5].

European ALS patients harboring *KIF5A* rare loss‐of‐function variants typically have a younger age at onset and prolonged survival [2], while very limited information is available for rs113247976. Our study aims to determine the frequency of the *KIF5A* rs113247976 in two Italian ALS cohorts and its impact on clinical variables associated with disease progression.

## 2 Methods

### 2.1 Participants and Clinical Assessment

We included 865 ALS Italian patients from the IRCCS Istituto Auxologico Italiano (discovery cohort) and 1174 from the Piemonte and Valle d'Aosta ALS Register (PARALS) (replication cohort) between 2013 and 2023 [6].

We recorded the following demographic and clinical data: sex, ALS family history, age at onset and at diagnosis, site of onset, survival after onset, and the ALSFRS‐R preslope at the first evaluation (calculated as (48—ALSFRS‐R score)/months between disease onset and evaluation). For the analysis of disease progression rate, we included only those patients for whom the first ALSFRS‐R score available had been assessed within 45 days from diagnosis.

### 2.2 Genetic Analyses

For the discovery cohort, SNP genotyping data, obtained from previous Human 660 W‐Quad BeadChips and Global Screening Arrays (Illumina) analyses [7], were used to impute the *KIF5A* rs113247976, as previously described [8]. For the replication cohort, whole‐genome sequencing data were generated as previously reported [9]. All patients were screened for mutations in the main four ALS‐associated genes (*C9orf72, SOD1, TARDBP* and *FUS*), as previously described [8].

### 2.3 Statistical Analyses

Statistical analyses were performed using RStudio version 2023.03. With no patients having the p.Leu986 homozygous genotype (TT), comparisons were made between the CC and CT genotypes. Cross‐tabulated frequencies were calculated using Fisher's exact tests. The Wilcoxon‐Mann–Whitney test was used for quantitative variables due to non‐normality (Shapiro–Wilk test). Multiple linear regression explored the impact of several variables on ALSFRS‐R preslope. The rs113247976 genotype's effect on survival was estimated using Kaplan–Meier and Cox regression analyses. Censoring was applied for patients alive at last follow‐up, and missing data were handled by pairwise deletion.

## 3 Results

Demographic and clinical features, and genotype data for the coding *KIF5A* rs113247976 (p.Pro986Leu) of the discovery (*n* = 875 patients) and replication (*n* = 1174 patients) Italian ALS cohorts are reported in Table 1. Compared to the discovery cohort, the replication one had a higher median age at onset by up to 6 years, longer survival by 6–7 years, more bulbar cases, more female patients, and a higher prevalence of familial cases. Additionally, more patients carried mutations in the four main ALS genes. No patients with the p.Leu986 variant in homozygous state were found, and both the ALSFRS‐R preslope and the minor allele frequency (MAF) of the p.Leu986 variant did not significantly differ between the two cohorts (Table 1). Further, rs113247976 MAF was similar (0.014 and 0.017) to previously described ALS populations from various European countries and the United States (0.02) [2]. For the replication cohort, additional rare (MAF < 0.1%) variants in minor ALS‐related genes and their frequency in p.Pro986 and p.Leu986 carriers are reported in Supporting Information S1 and Table S1.

**TABLE 1:** Comparison of demographic and clinical features and genotype data of the ALS discovery (*n* = 865) and replication (*n* = 1174) cohorts.

Variable	Discovery cohort		Replication cohort		*p*
	No patients (frequency)	Median (IQR)	No patients (frequency)	Median (IQR)	
Sex	865		1174		**1.18e‐05**
Male	554 (64.0%)		638 (54.3%)		
Female	311 (36.0%)		536 (45.7%)		
ALS family history	833		1170		**3.81e‐07**
FALS	20 (2.4%)		113 (9.6%)		
SALS	813 (97.6%)		1057 (90.0%)		
Mutations in common ALS genes	865		1174		**5.40e‐06**
*C9orf72*	20 (2.3%)		80 (6.8%)		
*SOD1*	0 (0.0%)		25 (0.2%)		
*TARDBP*	7 (0.8%)		16 (1.4%)		
*FUS*	2 (0.2%)		6 (0.5%)		
Age at onset (years)	798	61.6 (52.7–69.6)	1174	67.7 (59.7–74.1)	**1.93e‐26**
Survival from onset (months)	776	25.3 (13.1–46.1)	1174	32.7 (20.7–61.8)	**4.37e‐15**
Site of onset	865		1174		**8.55e‐05**
Bulbar	206 (23.8%)		374 (31.9%)		
Spinal	659 (76.2%)		800 (68.1%)		
ALSFRS‐R preslope^a^	449	0.60 (0.32–1.04)	1174	0.59 (0.29–1.16)	0.54
rs113247976 SNP genotype	865		1174		0.56
p.Pro986/p.Pro986	841 (97.2%)		1135 (96.7%)		
p.Pro986/p.Leu986	24 (2.8%)		39 (3.3%)		
MAF p.Leu986	0.014		0.017		0.57

Abbreviations: ALS, amyotrophic lateral sclerosis; ALSFRS‐R, ALS Functional Rating Scale Revised; FALS, familial ALS; IQR, interquartile range; MAF, minor allele frequency; no, number; SALS, sporadic ALS; SNP, single nucleotide polymorphism.

^a^ Only patients for whom the ALSFRS‐R score was assessed < 45 days from diagnosis were included. Significant *p* values (< 0.05) are reported in bold.

**FIGURE 1:**
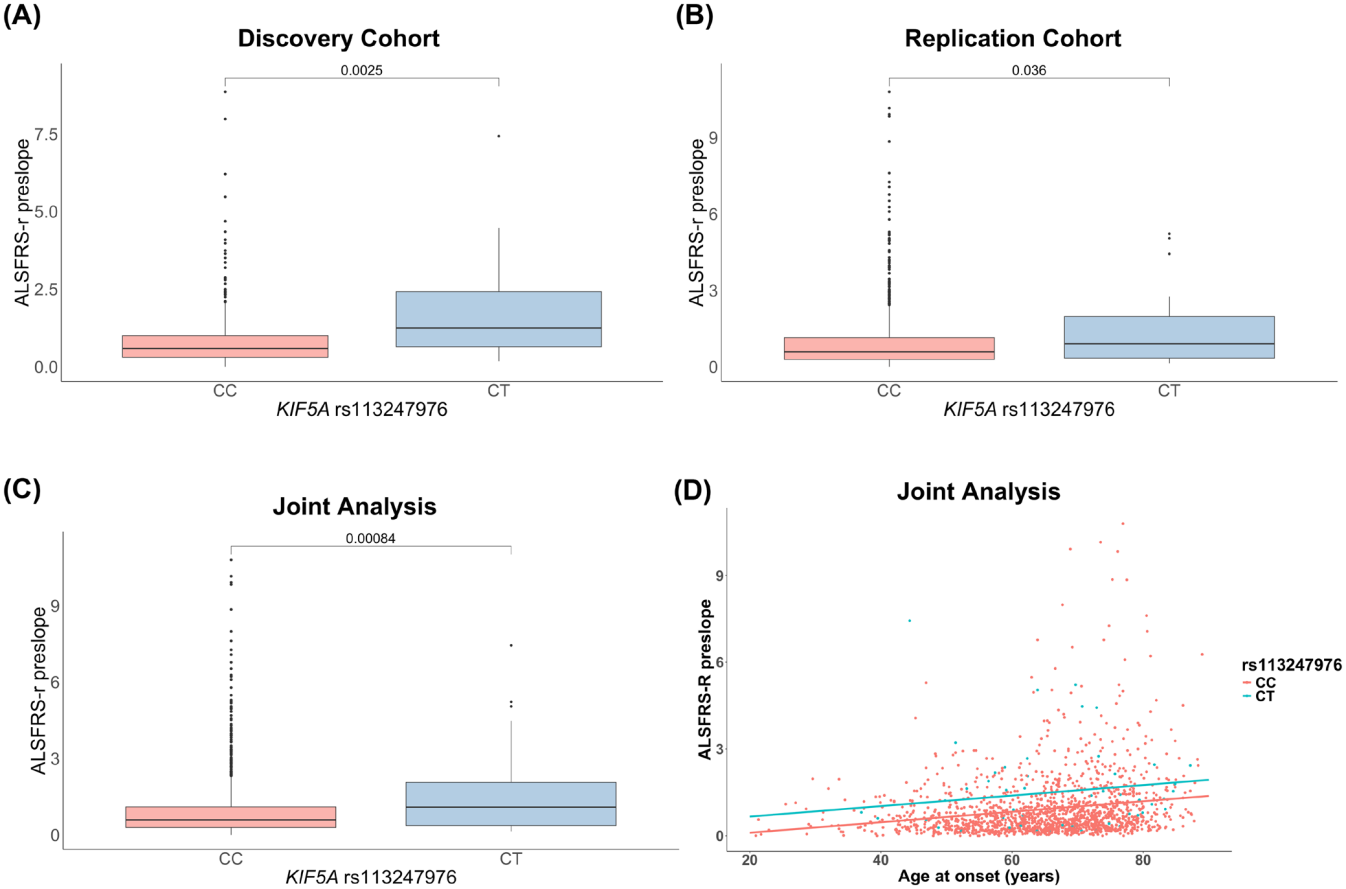
(A–C) Distribution of the ALSFRS‐R preslope according to the *KIF5A* rs113247976 genotypes (p.Pro986 and p.986Leu). (A) Discovery cohort (*n* = 449). (B) Replication cohort (*n* = 1174). (C) Aggregate analysis of discovery and replication cohorts (*n* = 1623). The non‐parametric Wilcoxon‐Mann–Whitney was run for the analysis. In the boxplots, the bold line shows the median, the gray box includes the middle 50% of the data and whiskers indicate the minimum and maximum values. Empty circles represent outliers. (D) Plot reflecting the multiple linear regression model predicting the preslope of the ALSFRS‐R using the rs113247976 *KIF5A* genotype and age at onset. The *x*‐axis represents age at onset; the *y*‐axis indicates the preslope of the ALSFRS‐R. The two lines (red and light blue) represent the regression relationship for the two different genotypes of the *KIF5A* rs113247976 (i.e., p.986Leu vs. p.Pro986). Data points show observed values of ALSFRS‐R preslope for corresponding ages at onset, differentiated by *KIF5A* rs113247976 genotype status.

In both cohorts, heterozygous p.Leu986 carriers showed a higher ALSFRS‐R preslope than homozygous p.Pro986 patients [discovery: 1.25 (IQR 0.65–2.42) vs. 0.59 (IQR 0.31–1.01), *p* = 0.0025; replication: 0.90 (IQR 0.34–1.98) vs. 0.59 (IQR 0.29–1.14), *p* = 0.036] (Figure 1A,B). Given the similar median values across cohorts, a combined analysis was performed, confirming this association [1.09 (IQR 0.37–2.06) vs. 0.59 (IQR 0.29–1.10), *p* = 0.00084] (Figure 1C).

Multiple linear regression showed that only the rs113247976 *KIF5A* genotype and age at onset significantly predicted the ALSFRS‐R preslope (*p* = 0.000246 and 3.51e‐14), unlike gender, onset site, and *C9orf72* status. After refining the model, it continued to effectively predict the ALSFRS‐R preslope [*F*(2, 1620) = 35.48; *p* = 8.323e‐16], despite explaining limited variance (adjusted *R* ^2^ = 0.04078) (Figure 1D; Table S2).

Conversely, we found no significant differences in the other demographic and clinical features collected, including survival after onset (Figure S1 and Table S3), between the p.Pro986/p.Pro986 and p.Pro986/p.Leu986 genotypes in both the discovery and replication cohorts, and their joint analysis.

## 4 Discussion

Our data support the involvement of the common *KIF5A* p.Leu986 variant, a known ALS risk factor, in influencing disease progression. Independently validated across discovery, replication, and combined cohorts, our findings show that p.Leu986 carriers experience faster early disease progression, as indicated by the ALSFRS‐R preslope. However, this early decline does not correlate with reduced survival, contradicting prior, statistically non‐significant suggestions of shorter disease duration in p.Leu986 carriers [5].

Noteworthy, among functional and disability scores, the ALSFRS‐R, which is widely recognized for its multidimensional approach to measuring functional impairment in ALS (i.e., bulbar, fine and gross motor domains, and respiratory function), has been shown to be significantly related to ALS outcome and prognosis [10].

Although the ALSFRS‐R score reflects functional decline, it does not consistently predict survival, as patients with the same score may have different affected domains, which can impact survival differently. This variability may partly explain the absence of survival differences despite faster disease progression associated with the p.Leu986 variant [11].

ALS progression is known to be influenced by a complex interplay of genetic and non‐genetic factors (i.e., age at onset, site of onset, psychosocial factors, body mass index) [12]. Regarding genetic determinants, repeat expansions in *C9orf72*, mutations in *FUS*, and intermediate‐length CAG repeats in *ATXN2* have been linked to faster disease progression [13, 14]. Conversely, individuals with either double mutations or the single p.D90A mutation in *SOD1*, as well as young patients with *ALS2* and *SETX*‐related autosomal recessive ALS, tend to experience a slower disease progression [13]. In addition, *ATXN2* patients exhibit more frequent spinal onset, concurrent frontotemporal dementia, and shorter survival up to 1 year [14]. Except for the *UNC13A* rs12608932 and the *CAMTA1* rs2412208 risk alleles, which influence shorter survival [8, 15], common variants have typically limited impact on progression outcomes (i.e., survival, age at onset). Notably, the simultaneous presence of multiple risk alleles (i.e., *UNC13A* rs12608932, *CAMTA1* rs2412208) or repeat expansions (i.e., *C9orf72*, *ATXN2*) is associated with significantly reduced survival [16]. Within this framework, our study underscores the importance of the *KIF5A* rs113247976 variant as a predictor of accelerated disease progression in early stages, thereby contributing valuable insights into the genetic modifiers of ALS.

However, a limitation of our study is that the discovery cohort comprises only Italian ALS patients from a single center, unlike the replication cohort, which comes from a prospective, population‐based registry in Northern Italy. This distinction accounts for the observed epidemiological and clinical discrepancies between the two cohorts. Nonetheless, the ALSFRS‐R preslope, measured within 45 days of diagnosis, and the MAF of the p.Leu986 variant were comparable across both cohorts, facilitating their comparative analysis. Moreover, the ALSFRS‐R score tends to decline curvilinearly rather than linearly, contradicting the assumption of a uniform progression rate [17]. This non‐linear pattern suggests that the initial ALSFRS‐R preslope might differ based on the time elapsed since onset. To mitigate potential bias introduced by assessing ALSFRS‐R scores at different disease durations, we focused our analysis on patients whose ALSFRS‐R evaluations were conducted within 45 days of diagnosis, in order to standardize the effect of disease duration on our results.

Overall, this research provides significant insights into the limited body of knowledge concerning the role of *KIF5A* in ALS progression. As ALS is highly heterogeneous, the elucidation of genetic and environmental determinants that influence its course is crucial for anticipating the potential success of therapeutic interventions. In different clinical trials, indeed, only a subset of patients responded to treatments, underscoring the need for patient stratification in future studies [18]. In this scenario, preliminary screening for variants associated with phenotypic traits in ALS, such as *KIF5A* rs113247976, which in our study is linked to an accelerated progression, could prove vital in forecasting the outcomes of treatment strategies and informing the selection of participants for forthcoming clinical trials and could enhance the power of the ENCALS Survival Prediction Model.

## Author Contributions

**Arianna Manini, Alberto Brusati, Antonia Ratti** and **Nicola Ticozzi:** conceptualization. **Arianna Manini, Rosario Vasta, Alberto Brusati, Davide Gentilini** and **Nicola Ticozzi:** methodology. **Arianna Manini, Rosario Vasta** and **Alberto Brusati:** formal analysis. **Arianna Manini, Rosario Vasta, Alessio Maranzano, Francesco Gentile, Stefano Messina, Federico Verde, Cristina Moglia** and **Nicola Ticozzi:** investigation. **Antonia Ratti, Vincenzo Silani, Adriano Chiò, John E. Landers, Bryan J. Traynor, Adriano Chiò** and **Nicola Ticozzi:** resources. **Arianna Manini, Rosario Vasta, Alberto Brusati, Alessio Maranzano, Francesco Gentile, Eleonora Colombo** and **Nicola Ticozzi:** data curation. **Arianna Manini:** writing – original draft preparation. **Arianna Manini, Rosario Vasta, Antonia Ratti** and **Nicola Ticozzi:** writing – review and editing. **Antonia Ratti, Vincenzo Silani** and **Nicola Ticozzi:** supervision. **Antonia Ratti** and **Nicola Ticozzi:** project administration. **Vincenzo Silani, Antonia Ratti** and **Nicola Ticozzi:** funding acquisition. All authors contributed to the article and approved the submitted version.

## Ethics Statement

The study was approved by the Ethics Committee of the IRCCS Instituto Auxologico Italiano (2021_05_18) and by the Ethics Committee of the Turin ALS Center (Comitato Etico Azienda Ospedaliero‐Universitaria Città della Salute e della Scienza, Torino). Written informed consent was obtained from each patient at the time of evaluation to use semi‐anonymized clinical data for research purposes. The study was conducted in accordance with the principles of the Declaration of Helsinki.

## Conflicts of Interest

V.S. received compensation for consulting services and/or speaking activities from AveXis, Cytokinetics, Italfarmaco, Liquidweb Srl, Novartis Pharma AG, and Zambon Biotech SA. He is on the Editorial Board of Amyotrophic Lateral Sclerosis and Frontotemporal Degeneration, European Neurology, American Journal of Neurodegenerative Diseases, Frontiers in Neurology, and Exploration of Neuroprotective Therapy. N.T. received compensation for consulting services from Amylyx Pharmaceuticals, Biogen, Italfarmaco, and Zambon Biotech SA. He is Associate Editor for Frontiers in Aging Neuroscience.

## Supporting information


**Supporting Information S1.** ALS‐related variants considered in the replication cohort.
**Table S1.** Comparison of the number of rare (MAF < 0.1%) variants in minor ALS‐related genes between carriers of the two *KIF5A* rs113247976 genotypes (p.Pro986 and p.986Leu).
**Table S2.** Multiple Linear Regression Model for the ALSFRS‐R preslope.
**Figure S1.** Univariate Kaplan–Meier survival analysis for the *KIF5A* rs113247976 genotypes (p.Pro986 and p.986Leu). (A) Joint analysis of discovery and replication cohorts (*n* = 1950). (B) Discovery cohort (*n* = 776). (C) Replication cohort (*n* = 1174).
**Table S3.** Multivariate Cox regression models including the presence of the *KIF5A* p.986Leu heterozygous mutation, age at onset, *C9orf72* mutational status, gender, and site of onset in the joint analysis (A), discovery cohort (B) and replication cohort (C). (B = unstandardized regression coefficient; SE B = standard error of the coefficient; Exp(B) = hazard ratio). *p* values < 0.05 are reported in bold.

## Data Availability

Anonymized raw data are archived on Zenodo (doi: 10.5281/zenodo.10953602) and are available upon request to the corresponding author.
