# Supplementary material for: KIF5A p.Pro986Leu Risk Variant and Accelerated Progression of Amyotrophic Lateral Sclerosis
Source: Ann Clin Transl Neurol. 2025 Apr 25;12(7):1499–503. doi: 10.1002/acn3.70059 (PMC12257138; doi:10.1002/acn3.70059)
Supplement: Supplementary file 1 — Supporting Information S1. ALS‐related variants considered in the replication cohort. Table S1. Comparison of the number of rare (MAF < 0.1%) variants in minor ALS‐related genes between carriers of the two KIF5A rs113247976 genotypes (p.Pro986 and p.986Leu). Table S2. Multiple Linear Regression Model for the ALSFRS‐R preslope. Figure S1. Univariate Kaplan–Meier survival analysis for the KIF5A rs113247976 genotypes (p.Pro986 and p.986Leu). (A) Joint analysis of discovery and replication cohorts (n = 1950). (B) Discovery cohort (n = 776). (C) Replication cohort (n = 1174). Table S3. Multivariate Cox regression models including the presence of the KIF5A p.986Leu heterozygous mutation, age at onset, C9orf72 mutational status, gender, and site of onset in the joint analysis (A), discovery cohort (B) and replication cohort (C). (B = unstandardized regression coefficient; SE B = standard error of the coefficient; Exp(B) = hazard ratio). p values < 0.05 are reported in bold. [file ACN3-12-1499-s001.docx]

**Supplementary Material** – **ALS-related variants considered in the replication cohort.**

- *ALS2*(NM020919):D543G, *ALS2*(NM020919):P1266R, *ALS2*(NM020919):T1472M;
- *ANG*(NM001097577):V129A;
- *ANO2*(NM001364791):A602T, *ANO2*(NM001364791):D437E, *ANO2*(NM001364791):D731Y, *ANO2*(NM001364791):K211T, *ANO2*(NM001364791):L596R, *ANO2*(NM001364791):Q22H, *ANO2*(NM001364791):Q405K, *ANO2*(NM001364791):R108W, *ANO2*(NM001364791):R240X, *ANO2*(NM001364791):R791W, *ANO2*(NM001364791):S235L, *ANO2*(NM001364791):S541F, *ANO2*(NM001364791):T297fs, *ANO2*(NM001364791):V642G;
- *ANXA11*(NM145868):159168del, *ANXA11*(NM145868):A58V, *ANXA11*(NM145868):D40G, *ANXA11*(NM145868):E397D, *ANXA11*(NM145868):G403A, *ANXA11*(NM145868):M34I, *ANXA11*(NM145868):R308X, *ANXA11*(NM145868):Y3H;
- *APEX1*(NM001641):A175V, *APEX1*(NM001641):G306C, *APEX1*(NM001641):G57C, *APEX1*(NM001641):L291fs, *APEX1*(NM001641):P261A, *APEX1*(NM001641):R237C;
- *C21ORF2*(NM004928):C265R, *C21ORF2*(NM004928):G101S, *C21ORF2*(NM004928):G244S, *C21ORF2*(NM004928):G248E, *C21orf2*:G248R, *C21ORF2*(NM004928):G248R, *C21ORF2*(NM004928):p.140140del, *C21ORF2*(NM004928):p.T234fs, *C21ORF2*(NM004928):P245delinsPVGREHGASQGP, *C21ORF2*(NM004928):R172Q, *C21ORF2*(NM004928):R278C, *C21ORF2*(NM004928):T46M, *C21ORF2*(NM004928):W294S, *C21ORF2*(NM004928):W294S;
- *CCNF*(NM001761):D662E, *CCNF*(NM001761):E125K, *CCNF*(NM001761):L106V, *CCNF*(NM001761):L47F, *CCNF*(NM001761):Q203L, *CCNF*(NM001761):S261P;
- *CEP112*(NM001199165):A109G, *CEP112*(NM001199165):C188R, *CEP112*(NM001199165):I101V, *CEP112*(NM001199165):Q521E;
- *CHCHD10*(NM213720):A35D;
- *CHMP2B*(NM014043):I29V, *CHMP2B*(NM014043):Q25R;
- *DAO*(NM001917):R38H;
- *DCTN1*(NM004082):A1234V, *DCTN1*(NM004082):E800G, *DCTN1*(NM004082):Q359X, *DCTN1*(NM004082):A354V, *DCTN1*(NM004082):K118E, *DCTN1*(NM004082):R532P;
- *ELP3*(NM018091):H261R, *ELP3*(NM018091):R192C, *ELP3*(NM018091):R245Q, *ELP3*(NM018091):S114G, *ELP3*(NM018091):T241S, *ELP3*(NM018091):V353A;
- *ERBB4*(NM005235):V986I;
- *ERLIN2*(NM007175):S79I;
- *FIG4*(NM014845):I880N, *FIG4*(NM014845):P703L, *FIG4*(NM014845):Q823X, *FIG4*(NM014845):Q884X, *FIG4*(NM014845):R283C, *FIG4*(NM014845):R381Q, *FIG4*(NM014845):Y18H;
- *GRN*(NM002087):K259N, *GRN*(NM002087):L255M, *GRN*(NM002087):T18A, *GRN*(NM002087):T327A, *GRN*(NM002087):T532N;
- *HNRNPA1*:F222L, *HNRNPA1*:G275V, *HNRNPA1*:G295R, *HNRNPA1*:N292K, *HNRNPA1*:Q127R;
- *KIF5A*(NM031157):A919fs, *KIF5A*(NM031157):E755K, *KIF5A*(NM031157):G623R, *KIF5A*(NM031157):L488R, *KIF5A*(NM031157):N1006S, *KIF5A*(NM031157):N973K, *KIF5A*(NM031157):R114X, *KIF5A*(NM031157):R712W, *KIF5A*(NM031157):R718W, *KIF5A*(NM031157):T767I, *KIF5A*(NM031157):V159L, *KIF5A*(NM031157):V516M;
- *LMNB1*(NM005573):A436S, *LMNB1*(NM005573):E301D, *LMNB1*(NM005573):K102N;
- *MAPT*(NM001377265):F8L, *MAPT*(NM001377265):G42S, *MAPT*(NM001377265):G49V, *MAPT*(NM001377265):R448X, *MAPT*(NM001377265):S346F;
- *MATR3*(NM018834):A422T, *MATR3*(NM018834):D123G, *MATR3*(NM018834):Q477H, *MATR3*(NM018834):R252K, *MATR3*(NM018834):S419L;
- *NEFH*(NM021076):G12R, *NEFH*(NM021076):G83R, *NEFH*(NM021076):H19N, *NEFH*(NM021076):K599T, *NEFH*(NM021076):L202delinsLARFAQ, *NEFH*(NM021076):L216P, *NEFH*(NM021076):S124G;
- *NEK1*(NM001199397):A150G, *NEK1*(NM001199397):A512V, *NEK1*(NM001199397):A545S, *NEK1*(NM001199397):D103E, *NEK1*(NM001199397):D26V, *NEK1*(NM001199397):D969Y, *NEK1*(NM001199397):D996V, *NEK1*(NM001199397):E1011Q, *NEK1*(NM001199397):E1194K, *NEK1*(NM001199397):E419D, *NEK1*(NM001199397):E420D, *NEK1*(NM001199397):H1168Y, *NEK1*(NM001199397):I1209fs, *NEK1*(NM001199397):I1220L, *NEK1*(NM001199397):I358M, *NEK1*(NM001199397):I961M, *NEK1*(NM001199397):L193F, *NEK1*(NM001199397):M1064I, *NEK1*(NM001199397):N704D, *NEK1*(NM001199397):N910fs, *NEK1*(NM001199397):Q377fs, *NEK1*(NM001199397):R127Q, *NEK1*(NM001199397):T1141I, *NEK1*(NM001199397):W409X;
- *OPTN*(NM001008212):E135X, *OPTN*(NM001008212):E322K, *OPTN*(NM001008212):I138T, *OPTN*(NM001008212):L111R, *OPTN*(NM001008212):L500P, *OPTN*(NM001008212):M573I, *OPTN*(NM001008212):Q530K, *OPTN*(NM001008212):Q547X, *OPTN*(NM001008212):Q89X, *OPTN*(NM001008212):R133K, *OPTN*(NM001008212):R215K, *OPTN*(NM001008212):R545Q, *OPTN*(NM001008212):R83C, *OPTN*(NM001008212):V306fs;
- *PFN1*(NM005022):E117G, *PFN1*(NM005022):G15R, *PFN1*(NM005022):V23M;
- *PRPH*(NM006262):D163Y, *PRPH*(NM006262):E342K, *PRPH*(NM006262):S408V409delinsSX, *PRPH*(NM006262):S408fs, *PRPH*(NM006262):V204E, *PRPH*(NM006262):Y460S, *PRPH*(NM006262):Y470S;
- *SETX*(NM015046):A1478T, *SETX*(NM015046):A1479V, *SETX*(NM015046):D1105G, *SETX*(NM015046):G1562D, *SETX*(NM015046):H476R, *SETX*(NM015046):I1644T, *SETX*(NM015046):I809V, *SETX*(NM015046):K2550R, *SETX*(NM015046):L393P, *SETX*(NM015046):M2229V, *SETX*(NM015046):P1464L, *SETX*(NM015046):R2414X, *SETX*(NM015046):S1860F, *SETX*(NM015046):S213F, *SETX*(NM015046):T2283fs, *SETX*(NM015046):T698I, *SETX*(NM015046):V919I, *SETX*(NM015046):Y21F;
- *SIGMAR1*(NM005866):A4V, *SIGMAR1*(NM005866):R133Q, *SIGMAR1*(NM005866):V82M;
- *SPAST*(NM014946):A89P, *SPAST*(NM014946):G259C, *SPAST*(NM014946):I129V, *SPAST*(NM014946):I153T, *SPAST*(NM014946):S302F;
- *SPG11*(NM025137):L1987S, *SPG11*(NM025137):M1272T, *SPG11*(NM025137):R2096H;
- *SPTLC1*(NM006415):C318S, *SPTLC1*(NM006415):G387A, *SPTLC1*(NM006415):G403E, *SPTLC1*(NM006415):R240C, *SPTLC1*(NM006415):V513A;
- *SQSTM1*(NM003900):D149E, *SQSTM1*(NM003900):G334E, *SQSTM1*(NM003900):M404V, *SQSTM1*(NM003900):P358L, *SQSTM1*(NM003900):P392l, *SQSTM1*(NM003900):P392L, *SQSTM1*(NM003900):P438L, *SQSTM1*(NM003900):V259L;
- *TAF15*(NM139215):G176R, *TAF15*(NM139215):G39E, *TAF15*(NM139215):p.512518del, *TAF15*(NM139215):R150K, *TAF15*(NM139215):S448I;
- *TBK1*(NM013254):D456N, *TBK1*(NM013254):E165D, *TBK1*(NM013254):E448fs, *TBK1*(NM013254):E695X, *TBK1*(NM013254):I393M, *TBK1*(NM013254):I443L, *TBK1*(NM013254):I87T, *TBK1*(NM013254):K567Q, *TBK1*(NM013254):R117X, *TBK1*(NM013254):R357Q, *TBK1*(NM013254):T210A, *TBK1*(NM013254):V421I, *TBK1*(NM013254):Y174C;
- *TIA1*(NM022173):P113S;
- *TUBA4A*(NM006000):A65fs, *TUBA4A*(NM006000):D438N, *TUBA4A*(NM006000):p.G410fs, *TUBA4A*(NM006000):T349S;
- *UBQLN2*(NM013444):A18V, *UBQLN2*(NM013444):G502D;
- *VAPB*(NM004738):P111L, *VAPB*(NM004738):R72G, *VAPB*(NM004738):T97A;
- *VCP*(NM007126):I590V, *VCP*(NM007126):R713G, *VCP*(NM007126):Y89H.

**Supplementary Table 1** – **Comparison of the number of rare (MAF <0.1%) variants in minor ALS-related genes between carriers of the two *KIF5A* rs113247976 genotypes (p.Pro986 and p.986Leu).**

|  | ***KIF5A*** **rs113247976 p.Pro986** | ***KIF5A*** **rs113247976 p.986Leu** |
| --- | --- | --- |
| *OPTN* | 18 (1.6%) | 0 (0%) |
| *TUBA4A* | 4 (0.4%) | 0 (0%) |
| *ANO2* | 16 (1.4%) | 1 (2.6%) |
| *C21ORF72* | 14 (1.2%) | 0 (0%) |
| *CCNF* | 6 (0.5%) | 1 (2.6%) |
| *SIGMAR1* | 4 (0.4%) | 0 (0%) |
| *SPAST* | 4 (0.4%) | 2 (5.1%) |
| *TBK1* | 16 (1.4%) | 0 (0%) |
| *ALS2* | 3 (0.3%) | 0 (0%) |
| *ANG* | 1 (0.1%) | 0 (0%) |
| *ANXA11* | 8 (0.7%) | 1 (2.6%) |
| *APEX1* | 6 (0.5%) | 0 (0%) |
| *CEP112* | 4 (0.4%) | 0 (0%) |
| *CHCHD10* | 1 (0.1%) | 0 (0%) |
| *CHMP2B* | 2 (0.2%) | 0 (0%) |
| *DAO* | 1 (0.1%) | 0 (0%) |
| *DCTN1* | 3 (0.3%) | 0 (0%) |
| *ELP3* | 3 (0.3%) | 0 (0%) |
| *ERBB4* | 1 (0.1%) | 0 (0%) |
| *ERLIN2* | 1 (0.1%) | 0 (0%) |
| *FIG4* | 7 (0.6%) | 0 (0%) |
| *GRN* | 4 (0.4%) | 0 (0%) |
| *HRNPA1* | 0 (0%) | 0 (0%) |
| *LMNB1* | 2 (0.2%) | 1 (2.6%) |
| *MAPT* | 5 (0.4%) | 0 (0%) |
| *MATR3* | 5 (0.4%) | 1 (2.6%) |
| *PRPH* | 6 (0.5%) | 0 (0%) |
| *SETX* | 15 (1.3%) | 0 (0%) |
| *SPG11* | 3 (0.3%) | 0 (0%) |
| *TAF* | 4 (0.4%) | 1 (2.6%) |
| *KIF5A* | 11 (1%) | 0 (0%) |

**Supplementary Table 2** – **Multiple Linear Regression Model for the ALSFRS-R preslope.**

| **ALSFRS-R preslope** | ***B*** | **95% CI for *B*** | | **SE *B*** | **R^2^** | **ΔR^2^** |
| --- | --- | --- | --- | --- | --- | --- |
|  |  | ***LL*** | ***UL*** |  |  |  |
| Model |  |  |  |  | 0.04 | 0.04*** |
| Constant | -0.258* | -0.57 | -0.05 | 0.159 |  |  |
| Age at onset | 0.018*** | 0.01 | 0.02 | 0.002 |  |  |
| rs113247976 *KIF5A* genotype | 0.558*** | 0.26 | 0.86 | 0.154 |  |  |

ALSFRS-R: Amyotrophic Lateral Sclerosis Functional Rating Scale Revised; *B*: regression coefficient; CI: confidence interval; SE: standard error; LL: lower limit; UL: upper limit; R^2^: coefficient of determination; ΔR^2^: adjusted R^2^.

* *p* < 0.05; ** *p* < 0.01; *** *p* < 0.001.

**Supplementary Figure 1 – Univariate Kaplan–Meier survival analysis for the *KIF5A* rs113247976** **genotypes (p.Pro986 and p.986Leu).** (**A**) Joint analysis of discovery and replication cohorts (n=1,950). (**B**) Discovery cohort (n=776). (**C**) Replication cohort (n=1,174).

**Supplementary Table 3** – **Multivariate Cox regression models including the presence of the *KIF5A* p.986Leu heterozygous ation, age at onset, *C9orf72* ational status, gender, and site of onset in the joint analysis (A), discovery cohort (B) and replication cohort (C).**

| (**A**) |  |  |  | **95% CI** | |  |
| --- | --- | --- | --- | --- | --- | --- |
|  | **B** | **SE B** | **Exp(B)** | **LL** | **UL** | ***p* value** |
| ***KIF5A* p.986Leu** | -0.085 | 0.273 | 0.919 | 0.538 | 1.569 | 0.76 |
| **Age at onset** | 0.021 | 0.004 | 1.022 | 1.013 | 1.029 | **2.81e-08** |
| ***C9orf72* pathogenic repeat expansion** | 0.334 | 0.218 | 1.397 | 0.911 | 2.142 | 0.13 |
| **Gender** | 0.220 | 0.089 | 1.246 | 1.047 | 1.482 | **0.01** |
| **Site of onset** | 0.274 | 0.103 | 1.315 | 1.074 | 1.610 | **0.008** |
|  |  |  |  |  |  |  |
| (**B**) |  |  |  |  |  |  |
|  | **B** | **SE B** | **Exp(B)** | **LL** | **UL** | ***p* value** |
| ***KIF5A* p.986Leu** | 0.137 | 0.342 | 1.147 | 0.872 | 0.586 | 0.69 |
| **Age at onset** | 0.039 | 0.005 | 1.040 | 0.962 | 1.030 | **<2e-16** |
| ***C9orf72* pathogenic repeat expansion** | 0.708 | 0.262 | 2.030 | 0.493 | 1.216 | **0.007** |
| **Gender** | 0.092 | 0.111 | 1.096 | 0.912 | 0.881 | 0.41 |
| **Site of onset** | 0.139 | 0.121 | 1.149 | 0.870 | 0.907 | 0.25 |
|  |  |  |  |  |  |  |
| (**C**) |  |  |  |  |  |  |
|  | **B** | **SE B** | **Exp(B)** | **LL** | **UL** | ***p* value** |
| ***KIF5A* p.986Leu** | 0.280 | 0.462 | 0.756 | 0.306 | 1.868 | 0.54 |
| **Age at onset** | 0.025 | 0.008 | 1.025 | 1.010 | 1.041 | **0.002** |
| ***C9orf72* pathogenic repeat expansion** | 0.871 | 0.407 | 2.389 | 1.076 | 5.302 | **0.03** |
| **Gender** | 0.148 | 0.150 | 1.159 | 0.864 | 1.555 | 0.32 |
| **Site of onset** | 0.082 | 0.207 | 1.086 | 0.723 | 1.631 | 0.69 |
|  |  |  |  |  |  |  |

CI: confidence interval; LL: lower limit; UL: upper limit; B = unstandardized regression coefficient; SE B = standard error of the coefficient; Exp(B) = hazard ratio.

*p* values <0.05 are reported in bold.
